# A Two-Photon Probe Based on Naphthalimide-Styrene Fluorophore for the *In Vivo* Tracking of Cellular Senescence

**DOI:** 10.1021/acs.analchem.0c05447

**Published:** 2021-01-27

**Authors:** Beatriz Lozano-Torres, Juan F Blandez, Irene Galiana, José A Lopez-Dominguez, Miguel Rovira, Marta Paez-Ribes, Estela González-Gualda, Daniel Muñoz-Espín, Manuel Serrano, Félix Sancenón, Ramón Martínez-Máñez

**Affiliations:** † Instituto Interuniversitario de Investigación de Reconocimiento Molecular y Desarrollo Tecnológico (IDM), Universitat Politècnica de València-Universitat de València, Camí de Vera S/ N, Valencia 46022 Spain; ‡ Unidad Mixta UPV-CIPF de Investigación en Mecanismos de Enfermedades y Nanomedicina, Universitat Politècnica de València, Centro de Investigación Príncipe Felipe, C/Eduardo Primo Yúfera 3, Valencia 46012, Spain; § CIBER de Bioingeniería, Biomateriales y Nanomedicina (CIBER-BBN), Av. Monforte de Lemos, 3-5. Pabellón 11. Planta 0, Madrid 28029, Spain; ∥ Unidad Mixta de Investigación en Nanomedicina y Sensores. Universitat Politècnica de València, IIS La Fe, Av. Fernando Abril Martorell, 10, Torre A 7a̲ planta, Valencia 46026, Spain; ⊥ Institute for Research in Biomedicine (IRB Barcelona), Barcelona Institute of Science and Technology (BIST), Carrer de Baldiri Reixac, 10, Barcelona 08028, Spain; # CRUK Cancer Centre Early Detection Programme, Department of Oncology, University of Cambridge, Hutchison/MRC Research Centre, Box 197, Cambridge CB2 0XZ, U.K.; ∇ Catalan Institution for Research and Advanced Studies (ICREA), Passeig Lluís Companys 23, 08010 Barcelona, Spain

## Abstract

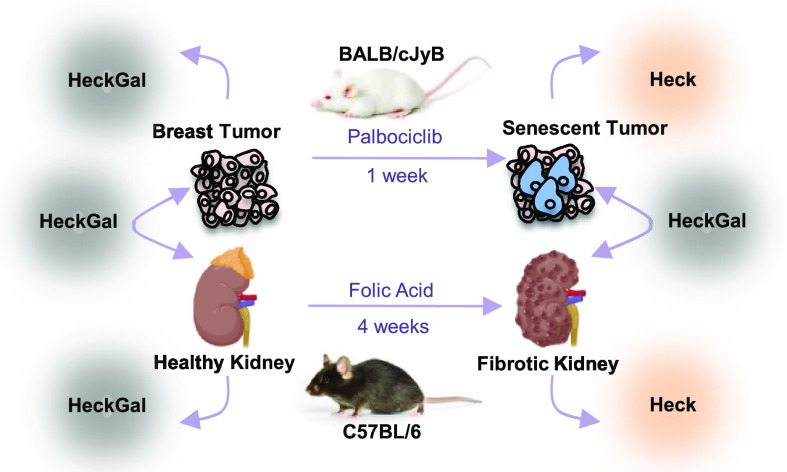

Cellular senescence is a state of stable cell cycle arrest that can negatively affect the regenerative capacities of tissues and can contribute to inflammation and the progression of various aging-related diseases. Advances in the *in vivo* detection of cellular senescence are still crucial to monitor the action of senolytic drugs and to assess the early onset or accumulation of senescent cells. Here, we describe a naphthalimide-styrene-based probe (**HeckGal**) for the detection of cellular senescence both *in vitro* and *in vivo*. **HeckGal** is hydrolyzed by the increased lysosomal β-galactosidase activity of senescent cells, resulting in fluorescence emission. The probe was validated *in vitro* using normal human fibroblasts and various cancer cell lines undergoing senescence induced by different stress stimuli. Remarkably, **HeckGal** was also validated *in vivo* in an orthotopic breast cancer mouse model treated with senescence-inducing chemotherapy and in a renal fibrosis mouse model. In all cases, **HeckGal** allowed the unambiguous detection of senescence *in vitro* as well as in tissues and tumors *in vivo*. This work is expected to provide a potential technology for senescence detection in aged or damaged tissues.

Cellular senescence is a biological process occurring in response to stress or damage and whose main role is to trigger tissue repair and to prevent the proliferation of stressed or damaged cells.^1^ Senescence triggered by excessive proliferation is known as “replicative senescence”,^2^ but senescence can also be triggered through diverse procedures, such as activation of oncogenes, inhibition of tumor suppressor genes, accumulation of DNA damage, the presence of reactive oxygen species (ROS), or nucleolar stresses among others. This type of senescence is known as stress-induced premature senescence (SIPS).^3^ Senescence has a relevant physiological role during development and promotes tissue regeneration in response to circumstantial damage, but the inefficient elimination of senescent cells during aging or upon persistent damage can produce inflammation, fibrosis, tissue aging, tumorigenesis, and metastasis.^1,4−6^

Evidence is accumulating that the selective elimination of senescent cells ameliorates a wide variety of aging-associated diseases, reverts long-term degenerative processes, and extends both lifespan and healthspan in mice.^7,8^ Inspired by these findings, there is a growing interest in developing drugs capable to induce apoptosis preferentially in senescent cells. In fact, senotherapies (treatments with senolytic or senomorphic drugs) are a new strategy to prevent cell-autonomous and non-cell-autonomous effects of senescent cells.^9−11^ Senolytic drugs kill senescent cells preferentially over nonsenescent cells; whereas, senomorphic drugs reduce the secretion of proinflammatory and profibrotic factors by senescent cells but without killing them.^12,13^ Such drugs would contribute to the therapeutic treatment of senescence-associated diseases and may stimulate the long-term idea that rejuvenation might be possible.^6^ A related important issue in the field of senotherapy is the development of new highly selective and sensitive tools to detect cellular senescence.^14^ These probes are expected to play an essential role in the detection of senescent cells in aged or damaged tissues, help in the discrimination between senolytic (selectively killing senescent cells) and senomorphic (selectively suppressing SASP) drugs, or monitor the action of senotherapeutics in multiple age-related disorders.^12,13^

Some of the most important markers of senescent cells are senescence-associated heterochromatic foci (SAHF),^15,16^ activation of tumor suppressors and cell cycle inhibitors (e.g., p53, p16INK4a, and p21),^17−20^ the overexpression of antiapoptotic proteins (e.g., BCLs),^21^ the absence of proliferative markers (Ki67),^22^ the loss of important chromatin structural proteins (Lamin B1, HMGB1, and HGMB2),^23^ a senescence-associated secretory phenotype (SASP),^24^ and the presence of high levels of lysosomal β-galactosidase (β-Gal) activity, known as senescence-associated β-galactosidase, (SA-β-Gal).^25^ Monitoring β-Gal activity using chromofluorogenic molecular-based probes represents a simple and accurate manner to track senescence in most of the cases, and several β-Gal probes are commercially available. However, most of these probes cannot be applied to *in vivo* models.^12,13^ For example, fluorescein-di-(β-D-galactopyranoside) (FDG) requires the use of chloroquine in order to increase lysosomal pH,^26,27^ 4-methylumbelliferyl β-D-galactopyranoside (MUG) is not permeable to cells,^28^ and 5-bromo-4-chloro-3-indolyl-β-D-galactopyranoside (X-Gal)^29^ is toxic and therefore cells need to be previously fixed. These drawbacks have boosted the interest in developing molecular sensors for the monitoring of β-Gal activity that could work on live cells and tissues. More recently, ((*E*)-2-(2-(6-hydroxy-2,3-dihydro-1*H*-xanthen-4-yl)vinyl)-3,3-dimethyl-1-propyl-3*H*-indol-1-ium), known as Spider-Gal, has gained importance as an SA-β-Gal detection kit, especially for flow cytometry since the fluorophore is covalently anchored to the cell after hydrolysis. However, this could be a problem when applying *in vivo* and cause long-term toxicity.^30^ Most of the described probes are based on classical one-photon fluorophores linked to the anomeric carbon of β-galactose. In one-photon probes, the biological target is only detected by an intensity-responsive fluorescent signal, which can be interfered with the excitation and emission efficiency, probe concentration, and surrounding conditions.^31,32^ As an alternative, the design of molecular probes using two-photon fluorophores has attracted great attention in the last years due to their improved three-dimensional spatial localization, prolonged observation time, increased imaging depth, minimized fluorescence background and light scattering, and lower tissue injury.^33^ In addition, one typical inaccuracy when developing SA-β-Gal probes is their validation in biological models, which is not directly related to senescence. In most cases, the method used in previous *in vitro* studies are based on *lac*Z gene transfection and *in vivo* using mouse models in which tumors are labelled with avidin-β-Gal^34,35^ or transfected with the pCMV-*lac*Z plasmid^36^ that results in high levels of cytosolic β-Gal expression,^37−44^ which is unrelated to the lysosomal human β-Gal, encoded by the GLB1 gene, naturally overexpressed in senescent cells. As a consequence, there are still a very limited number of selective two-photon fluorescent probes for the detection of bona fide cellular senescence *in vivo* models. Specifically, SG1 was the first two-photon ratiometric probe to detect senescent cells *in vitro*,^45^ and AHGa was the first two-photon probe to detect cellular senescence *in vivo*.^46^

Taking into account our interest in the development of fluorogenic sensors,^47−51^ we report herein the synthesis and characterization of a new two-photon naphthalimide-styrene probe (**HeckGal** in Figure 1A) for *in vivo* detection of senescence. The **HeckGal** probe consists of a naphthalimide-styrene fluorophore (**Heck** in Figure 1A) covalently linked to an acetylated β-galactose through the anomeric carbon. **HeckGal** is poorly emissive, whereas a sudden revival of the emission is observed in the presence of lysosomal β-Gal activity. The probe is tested *in vitro* in human cancer cell lines, including SK-Mel-103 and A549 cells, a breast murine cancer line (4 T1), and in a human BJ fibroblast cell line, undergoing senescence by different triggers. The probe is also tested *in vivo* in BALB/cByJ female mice bearing 4 T1 breast cancer tumors treated with senescence-inducing chemotherapy, and in a model of renal fibrosis induced by treatment with folic acid in C57BL/6 J male mice.

**Figure 1:**
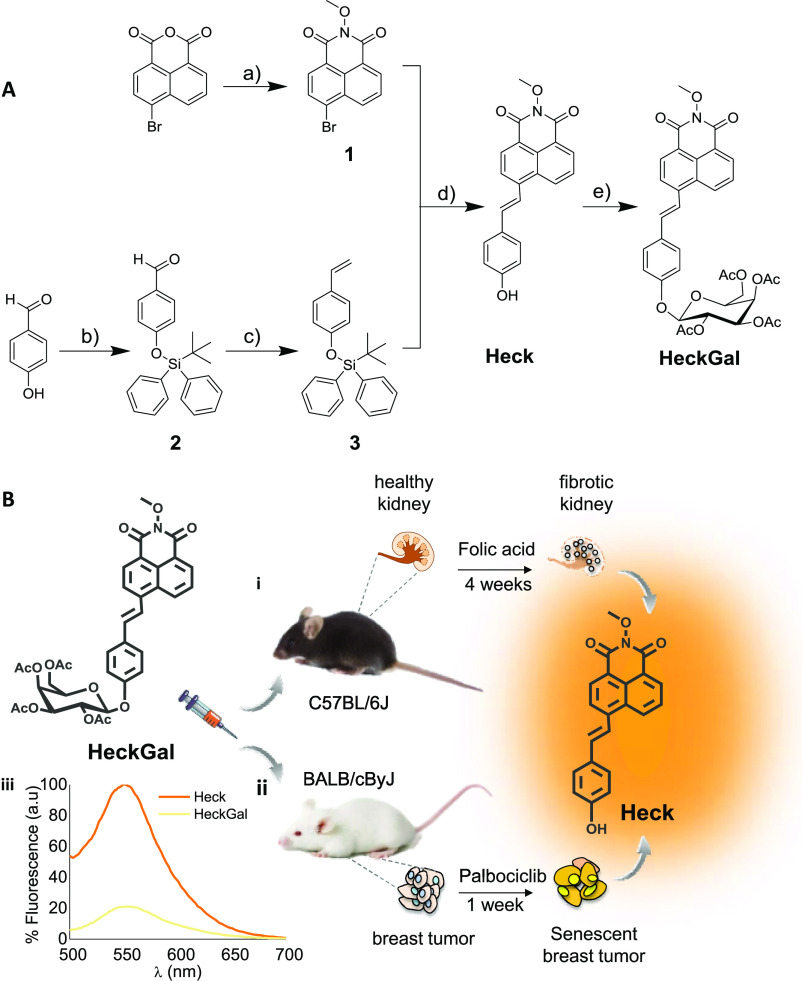
Synthesis of the probe and the mechanism of action in mice. (A) Synthetic route used for the preparation of the probe: (a) CH_3_ONH_2_·3HCl, Et_3_N, and dioxane; (b) TBDPSCl, imidazole, and DMF; (c) *n*-BuLi, Ph_3_PCH_3_I, and THF; (d) Pd(OAc)_2_, (*O*-tolyl)_3_P, Et_3_N, and DMF; and (e) NaOH/MeOH and acetobromo-α-D-galactose. (B) Schematic representation of the application of the probe in two *in vivo* models of senescence: (i) kidney fibrotic C57BL/6 J male mice induced by treatment with folic acid and (ii) BALB/cByJ female mice bearing 4 T1 breast cancer tumors treated with senescence-inducing chemotherapy. (iii) Fluorescence emission spectra (λ_ex_ = 488 nm) of **HeckGal** (yellow) and **Heck** fluorophore (orange) in aqueous solutions (pH 7)–DMSO (0.01%).

## Experimental Section

### Materials

All chemical reagents were purchased from Sigma–Aldrich, while anhydrous solvents and phosphate-buffered saline (PBS, 0.01 M) were purchased from Scharlab S.L. and used without further purification. Palbociclib was purchased from Selleckchem, and Dulbecco’s modified Eagle medium (DMEM) and fetal bovine serum (FBS) were purchased from Gibco. Flat-bottom clear 96-well plates were purchased from Promega. High-resolution mass spectrometry (HRMS) and the data was recorded with a TripleTOF T5600 (ABSciex, U.S.A.) spectrometer. ^1^H and ^13^C NMR spectra were collected on a Bruker FT-NMR Avance 400 (Ettlingen, Germany) spectrometer at 300 K, using TMS as an internal standard. HPLC measures were obtained by a Waters 1525 binary HPLC pump, and spectra were recorded by a Waters 2998 photodiode array at 260 nm. Fluorescence spectra were recorded by a JASCO FP-8500 fluorescence spectrophotometer, Luminescence was collected in a VICTOR multilabel plate reader (PerkinElmer). Confocal fluorescence images were taken on a Leica TCS SP8 AOBS, and two-photon images were acquired using a multiphoton Olympus FV1000MPE confocal microscope. Images were analyzed using ImageJ software. The SK-Mel-103 (human melanoma) cancer cell line and 4 T1 (breast cancer cells) were acquired from the American Type Culture Collection (ATCC). BALB/cByJ female mice were purchased from Charles River laboratories, France.

### Hydrolysis Reaction

The hydrolysis reaction of the **HeckGal** probe by β-Gal enzyme was analyzed by fluorescence spectroscopy and by HPLC–UV techniques. For this purpose, 2 μL of human β-Gal enzyme was added to PBS (pH 7)–DMSO (0.01%) solutions of **HeckGal** (10^–5^ M), and the emission spectrum at 552 nm was recorded with time (Figure S7a). After 15 min, **HeckGal** was completely hydrolyzed, and the emission band of the product closely correlated with the emission intensity of pure **Heck** fluorophore solution. Furthermore, in the same reaction condition, HPLC–UV studies (Figure S7b) corroborated these results. Time-conversion plots of **HeckGal** and its reaction intermediates (**Heck** and β-Gal) were determined by analyzing reaction aliquots by reversed-phase liquid chromatography using a KromasilC18 column as the stationary phase, eluting under isocratic conditions 0.8 mL/min (87.4:12.5:0.1 vol % H_2_O/CH_3_CN/CH_3_COOH) and using a photodiode array detector. Retention time (Rt) for **Heck** was 18.17 min, while Rt for **HeckGal** was 8.55 min and 4.60 min for human β-Gal enzyme.

### Cell Lines

SK-Mel-103 (human melanoma) cancer cells and 4 T1 (mouse breast cancer cells) were obtained from ATCC. Cells were maintained in a DMEM supplemented with 10% FBS and incubated in 20% O_2_ and 5% CO_2_ at 37 °C. Cells were routinely tested for mycoplasma contamination using the mycoplasma tissue culture NI (MTC-NI) rapid detection system (Gen-Probe). For senescence induction, cells were supplemented for 2 weeks with media containing 5 μM palbociclib.

### *In Vitro* Viability Assays

SK-Mel-103 (human melanoma) cancer and 4 T1 (mouse breast cancer) cells were used for cell viability assays. Cells were maintained in a DMEM supplemented with 10% FBS and incubated in 20% O_2_ and 5% CO_2_ at 37 °C. For senescence induction, cells were supplemented with a DMEM containing 5 μM palbociclib for 2 weeks. Control and senescent cells were placed in flat-bottom-clear 96-well plates at a density of 6000 and 4000 cells per well, respectively. The following day cells were treated with serial dilutions of **HeckGal** or **Heck**. Viability was assessed 48 h later with CellTiter-GLO luminescent cell viability assay. Raw data were obtained by measuring luminescence in a VICTOR multilabel plate reader (PerkinElmer).

### Mouse Models

Balb/cByJ mice were maintained at the Spanish Research Centre Principe Felipe (CIPF) in accordance with the recommendations of the Federation of European Laboratory Animal Science Associations (FELASA). Breast 4 T1 tumors were established by using 4 T1 cells. Cells were routinely cultured in a DMEM supplemented with 10% FBS and penicillin–streptomycin. In order to generate breast tumors, cells were trypsinised, counted with a LUNA automated cell counter, and injected subcutaneously in the left breast of 28 to 34 week-old BALB/cByJ female mice at a concentration of 0.5 × 10^6^ cells in a volume of 100 μL. Tumor volume was measured every 2 days with a caliper and calculated as *V* = (*a* × *b*^2^)/2 where *a* is the longer and *b* is the shorter of two perpendicular diameters. Palbociclib or vehicle was administered by daily oral gavage for 7 days with 100 mg/kg dissolved in 50 mM sodium lactate, at pH 5 in order to induce senescence. Then, **HeckGal** was intraperitoneally (i.p.) administered at a concentration of 6 mg/mL in a DMEM (5% DMSO) in a volume of 200 μL. Mice were sacrificed 2 h later by CO_2_ exposure in a euthanasia chamber, and tumors and organs (lung, liver, kidney, or spleen) were immediately removed. Tumors and organs were analyzed immediately after harvesting. **Heck** was detected using an excitation wavelength of 500 nm and an emission wavelength of 540 nm. Fluorescence images were taken on an IVIS spectrum imaging system and analyzed by using living imaging software from Caliper Life Sciences. On the other hand, 2 month-old C57BL/6 J male mice were maintained at the Institut de Recerca Biomèdica (IRB). All animal procedures were carried out in compliance with the regulations of the Animal Care and Use Ethical Committee of the Barcelona Science Park (CEEA-PCB) and the Catalan Government under the recommendations of the FELASA. In order to generate renal fibrosis, mice were i.p injected with a single dose of either 250 mg/kg of folic acid or vehicle. Thirty-four days after treatment, the animals were administered either with a single i.p injected dose of **HeckGal** (13.33 mg/mL 200 μL) in DMSO 1% corn oil or with vehicle. Animals were euthanized 5 h later by CO_2_ exposure in a euthanasia chamber, and the kidneys were excised for observation with an IVIS imager (PerkinElmer).

### Preparation of Mouse Tumor Slices for Imaging Experiments

Tumors from Balb/cByJ mice ortothopically injected with 4 T1 cells treated or not treated with palbociclib were excised and cut in half. They were pasted onto a petri dish exposing a tumor surface as smooth as possible. The slices were incubated with a 10 mM solution of **HeckGal** for 2 h at 37 °C in a dry incubator, and then washed three times with PBS and observed under a two-photon confocal microscope (Olympus FV1000MPE). The images were acquired at different penetration depths (λ_ex_ = 820 nm).

## Results and Discussion

### Synthesis, Characterization, Spectroscopic Features, and the Mechanism

The **HeckGal** probe was synthesized following the synthetic procedure shown in Figure 1A. Naphthalimide **1** was obtained by the reaction between 4-bromo-1,8-naphthalic anhydride and methoxylamine in refluxing dioxane. In parallel, the hydroxyl group of 4-hydroxybenzaldehyde was protected with *t*-butylchlorodiphenylsilane (TBDPSCl) yielding compound **2**, in which the aldehyde was converted into a double bond using a Wittig reaction resulting in compound **3**. A Heck cross-coupling reaction between compounds **1** and **3** yielded **Heck** fluorophore. Finally, **Heck** was consecutively reacted with NaOH, in order to remove the phenolic proton, and with 2,3,4,6-tetra-*O*-acetyl-α-D-galactopyranosyl bromide (Gal) yielding the **HeckGal** probe. The final probe and intermediate compounds were fully characterized by ^1^H NMR, ^13^C NMR, and HRMS (Figures S1–S5). PBS (pH 7)–DMSO (0.01%) solutions of the **Heck** fluorophore (10^–5^ M) presented an intense emission band centered at 550 nm (Φ**_Heck_** = 0.875) when excited at 488 nm (Figure 1B (iii)). In contrast, excitation at 488 nm of PBS (pH 7)–DMSO (0.01%) solutions of **HeckGal** resulted in a weak broad emission (Φ**_HeckGal_** = 0.074) (Figure 1B (iii)). The low emission intensity of **HeckGal**, when compared to that of **Heck**, is ascribed to a photoinduced electron transfer process from the galactose unit to the excited fluorophore. It was also assessed that the emission intensity of **Heck** remained unchanged in the 4–9 pH range (Figure S6). After assessing the photophysical properties, time-dependent fluorescent measurements in PBS (pH 7)–DMSO (0.01%) solutions of **HeckGal** in the presence of β-Gal were carried out (Figure S7A). Progressive enhancement of the emission at 550 nm was observed due to the generation of free **Heck** produced by the enzyme-induced hydrolysis of the *O*-glycosidic bond in **HeckGal**. The reaction was also analyzed by HPLC (Figure S7B), which showed the progressive vanishing of the **HeckGal** peak (at ca. 8.5 min) with the subsequent appearance of the **Heck** signal at ca. 8.2 min.

**HeckGal** displays several advantages when compared with the recently reported AHGa probe. **HeckGal** presents a more extended conjugated framework that is reflected in a marked increase, of almost 100 nm, in the two-photon excitation wavelength. This increase in excitation wavelength might allow greater tissue penetrability, less phototoxicity, and reduced light scattering. Moreover, the molecule generated after **HeckGal** hydrolysis with β-Gal enzyme (i.e., the **Heck** fluorophore) shows a remarkable higher quantum yield of 0.875, making the **HeckGal** probe more suitable for the differentiation between senescent and nonsenescent cells with high basal levels of the β-Gal enzyme. In addition, a comparative table of **HeckGal** and other cell senescence probes published in the last 3 years is shown in the Supporting Information (Table S1).

### *In Vitro* Validation of the HeckGal Probe

**Figure 2:**
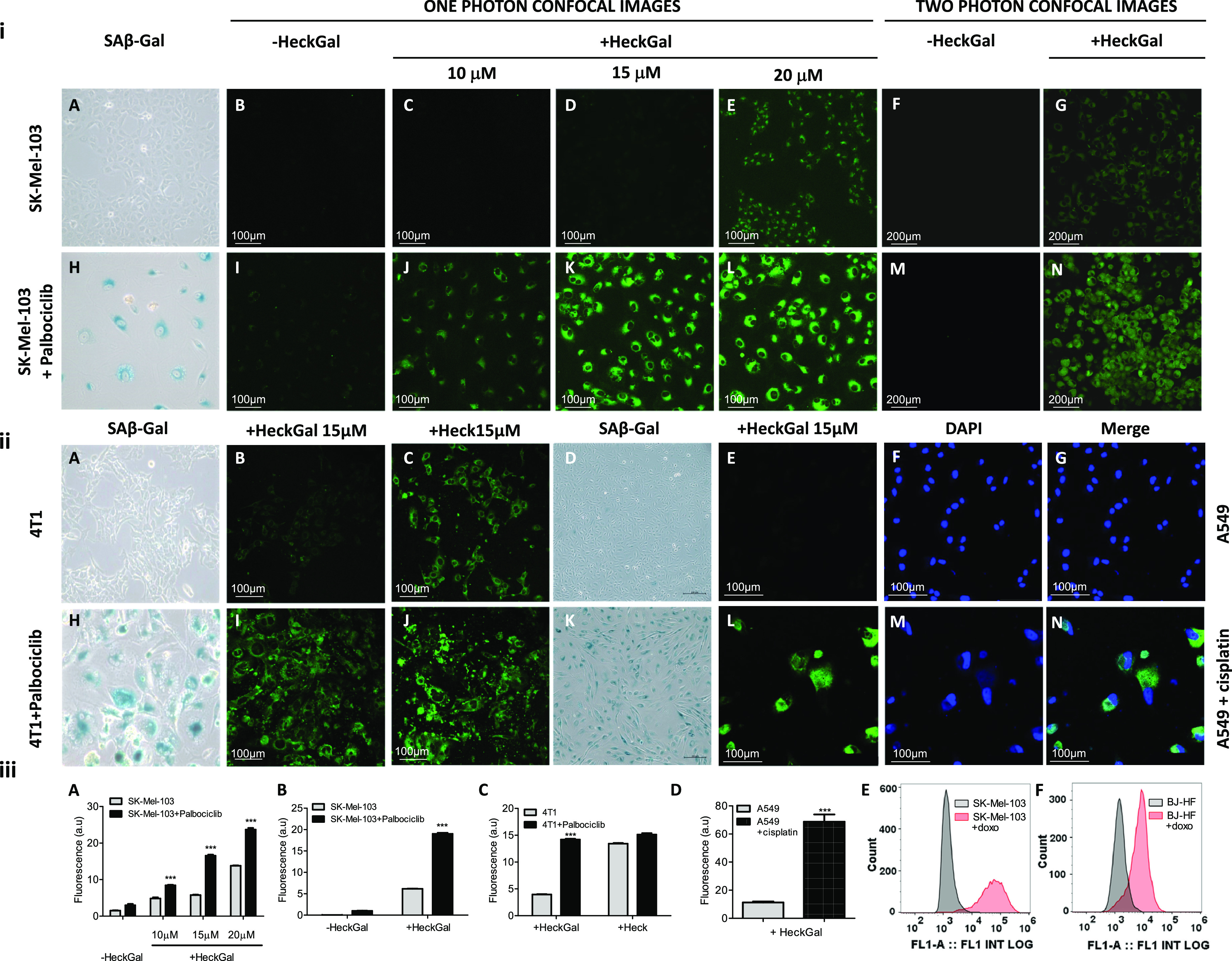
Probe enables the detection of senescence in various cell lines regardless of the induction method. (i) Senescence induction was assessed by SA-β-Gal staining in (A) nontreated and (H) palbociclib-treated cells. Note that senescent SK-Mel-103 cells present the typical blue staining. Confocal images of SK-Mel-103 and SK-Mel-103 treated with palbociclib. (B–E and I–L) One-photon confocal images of (B–E) control SK-Mel-103 in the (B) absence or (C–E) presence of 10, 15, and 20 μM of a **HeckGal** probe, respectively, and (I–L) SK-Mel-103 treated with palbociclib in the (I) absence or (J–L) presence of 10, 15, and 20 μM of the **HeckGal** probe, respectively. (F, G, M, N) Two-photon microscopy images of (F, G) nontreated and (M, N) palbociclib-treated (senescent) SK-Mel-103 cells in (F, M) the absence and (G, N) presence of 10 μM of the **HeckGal** probe. Cells were incubated with **HeckGa**l in a DMEM (10% FBS, 0.1% DMSO) in 20% O_2_ and 5% CO_2_ at 37 °C for 2 h, and then one-photon images were acquired by using a confocal microscope (Leica TCS SP8 AOBS), and two-photon images were acquired by using a multiphoton confocal microscope (Olympus FV1000MPE). (ii) SA-β-Gal staining of (A) nontreated and (H) palbociclib-treated 4 T1 cells. Note that senescent 4 T1 cells present the typical blue staining. (B, C, I, J) Confocal images of (B, C) control 4 T1 cells in the presence of (B) 15 μM of the **HeckGal** probe or (C) 15 μM of **Heck** and 4 T1 cells treated with (I, J) palbociclib in the presence of (I) 15 μM of the **HeckGal** probe or (J) 15 μM of **Heck**. SA-β-Gal staining of (D) nontreated and (K) cisplatin-treated A549 cells. Note that senescent A549 cells present the typical blue staining. Confocal microscopy images of (E−G) nontreated and (L–N) cisplatin-treated A549 cells, exposed to the **HeckGal** probe. Cells were incubated with **HeckGal** (15 μM) in a DMEM + 10% FBS in 20% O_2_ and 5% CO_2_ at 37 °C for 2 h, and images were acquired by using a confocal microscope (excitation at 488 nm). (iii) Quantification of the fluorescence emission intensity relative to the cell surface of control and palbociclib-treated SK-Mel-103 cells incubated with **HeckGal** visualized with (A) one-photon confocal imaging and (B) two-photon confocal imaging. Quantification of the fluorescence emission intensity relative to the cell surface of control and palbociclib-treated 4 T1 cells incubated with **HeckGal** or **Heck** visualized with (C) one-photon confocal imaging. Quantification of the fluorescence emission intensity relative to the cell surface of control and cisplatin-treated A549 cells incubated with **HeckGal** visualized with (D) one-photon confocal imaging. Error bars represent SEM (*n* = 3). (E) Fluorescence-activated cell sorting (FACS) analysis for control SK-Mel-103 (gray) human melanoma cells and doxorubicin-treated SK-Mel-103 (red) cells after treatment with **HeckGal**. (F) FACS analysis for control BJ (gray) human fibroblast cells and doxorubicin-treated BJ (red) cells after treatment with **HeckGal**. Both cell lines were treated with 250 nM doxorubicin for 24 h in order to induce cellular senescence, or with DMSO as the vehicle. After 14 days, upon complete development of the senescent phenotype, cells were incubated with 7 μM **HeckGal** for 2 h, detached from the plates, and washed twice with PBS. **HeckGal** fluorescence was subsequently evaluated by a Sony SA3800 spectral analyzer.

To study the cellular toxicity after prolonged exposure to the **HeckGal** probe, human melanoma SK-Mel-103 and murine breast cancer 4 T1 cells were used in cell viability assays, and the results showed that after 48 h, neither **Heck** nor **HeckGal** were toxic for SK-Mel-103 or 4 T1 cells, in both senescence and nonsenescence states, at concentrations of up to 100 μM (Figure S8). Once proven the probe’s biocompatibility, the preferential activation of **HeckGal** in senescent cells *in vitro* was assessed in senescent SK-Mel-103, 4 T1, A549 (human lung carcinoma), and BJ (human fibroblast) cell lines. Senescence was induced in SK-Mel-103 and 4 T1 cells by treatment with 5 μM palbociclib, a well-known specific CDK4/6 inhibitor,^52^ for 2 weeks. After palbociclib treatment, the cell morphology changed, presenting an enlarged and flattened appearance typical of cellular senescence. Cellular senescence was assessed by SA-β-Gal activity assay (Figure 2i (A,H), 2ii (A,H)). Next, control and senescent SK-Mel-103 cells were seeded in flat-bottom-clear 96-well plates and incubated with 10, 15, and 20 μM solutions of **HeckGal** in a DMEM (0.1% DMSO) for 2 h in the case of one-photon studies. In the case of two-photon studies, cells were seeded in 96-well plates and incubated with a 10 μM solution of the probe. Cells were imaged by confocal microscopy using an excitation wavelength of 488 nm and by two-photon confocal microscopy using a 950 nm excitation wavelength. Control (Figure 2i (B,F)) and senescent (Figure 2i (I,M)) SK-Mel-103 cells did not show significant background signals before incubation with **HeckGal**, especially in two-photon studies (compare panels I and M in Figure 2i). Nonsenescent SK-Mel-103 cells showed weak emission in the presence of increasing concentrations (10, 15, and 20 μM) of the **HeckGal** probe (Figure 2i (C–E,G)), while palbociclib-treated SK-Mel-103 cells displayed an intense fluorescent signal that increased for higher **HeckGal** concentrations (Figure 2i (J–L,N)). The fluorescent signal in the cells is attributed to the hydrolysis of **HeckGal** into the **Heck** fluorophore that occurred preferably in senescent cells, which presents an increased β-galactosidase activity. Moreover, the emission spectrum of **Heck**, obtained after two-photon excitation (Figure S9), corresponds to that obtained in a fluorimeter when using one-photon 488 nm excitation wavelength (Figure 1B (iii)). Fluorescence quantification from the confocal images associated with each treatment showed a fluorescence enhancement (ca. 2.9-fold) in palbociclib-treated SK-Mel-103 cells incubated with 15 μM of the probe in one-photon confocal images (Figure 2iii (A)) and ca. 3.1-fold for cells incubated with 10 μM of the probe in two-photon images (Figure 2iii (B)). Moreover, the ability of **HeckGal** to detect senescent 4 T1 cells was also confirmed. Nontreated and palbociclib-treated (senescent) 4 T1 cells were incubated with 15 μM solutions of **HeckGal** or **Heck** in a DMEM (0.1% DMSO) for 2 h. Figure 2ii shows that control 4 T1 cells treated with **HeckGal** (Figure 2ii (B)) showed a minimal fluorescence when compared to senescent 4 T1 cells (Figure 2ii (I)) in the same conditions (3.6-fold enhancement, Figure 2iii (C)). This marked difference was not observed when control and senescent 4 T1 cells were treated with **Heck** (Figure 2ii (C,J)), demonstrating the selectivity of **HeckGal** to detect cellular senescence. The versatility of the **HeckGal** probe was also validated in other cell lines where senescence was induced with different chemotherapies. Thus, human lung adenocarcinoma (A549) cells were treated with cisplatin (15 μM) for 3 weeks. Further incubation with **HeckGal** resulted in an enhanced fluorescence (ca. 6.1-fold, see Figure 2iii (D) for quantification of images) in cisplatin-treated A549 cells when compared with nontreated A549 cells (Figure 2ii (E,L)). Finally, co-staining with typical staining kits did not affect the **Heck** fluorescence signal or hydrolysis of **HeckGal** (Figure S10). The use of the **HeckGal** probe was also assessed by fluorescence-activated cell sorting (FACS) (Figure 2iii (E,F)) For these studies, control SK-Mel-103 cells and BJ human fibroblasts (gray) were exposed to 250 nM doxorubicin for 24 h to induce cellular senescence (red). On day 14, control and senescent cells from both cell lines were treated with 7 μM solutions of **HeckGal** for 2 h, detached from the plates, and fluorescence was subsequently evaluated through FACS. The studies demonstrated that **HeckGal** can distinguish between control and senescent cell populations in doxorubicin-induced SK-Mel-103 and BJ human fibroblasts.

### *In Vivo* Validation of the HeckGal Probe

**Figure 3:**
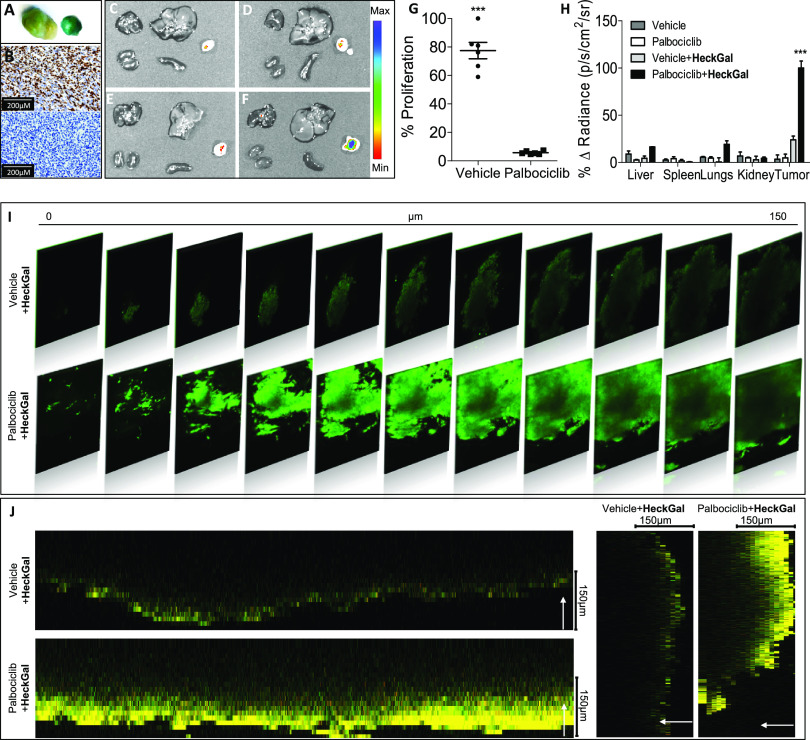
**HeckGal** probe enables the detection of senescence in different disease models of senescence. (A) Representative images of tumors stained for the SA-β-Gal assay: tumors from vehicle (left) and palbociclib-treated mice (right). (B) Immunohistochemical detection of the proliferation marker Ki67 in paraffin sections of tumors from vehicle (top) and palbociclib-treated mice (bottom). (C–F) IVIS images of organs and tumors from BALB/cByJ female mice bearing 4 T1 breast cancer cells: From left to right and from top to bottom: lungs, liver, tumor, kidney, and spleen; (C) Vehicle mice, (D) vehicle mice treated with (13.33 mg/mL, 100 μL), (E) mice treated with palbociclib for 1 week, (F) palbociclib-treated mice injected with **HeckGal** (13.33 mg/mL, 100 μL). Mice were sacrificed 2 h post-**HeckGal** treatment. (G) Quantification of the Ki67 signal in paraffin sections of tumors from vehicle (top) and palbociclib-treated mice (bottom). Error bars represent s.d. (H) Quantification of average radiance intensity from organs and tumors showed in images (C), (D), (E), and (F). Error bars represent SEM (*n* = 3 for each condition). (I) Two-photon fluorescence depth images of **HeckGal** in tumor tissue slices from vehicle (up) and palbociclib-treated mice (down). The slices were incubated with **HeckGal** (10 mM) for 2 h at 37 °C in a dry incubator. The images were acquired at different penetration depths (λ_ex_ = 820 nm). (J) 3D representation of images shown in Figure 3I demonstrating the greater penetrability of **HeckGal** in tumor tissue slices from palbociclib-treated mice (down) compared to tumor tissue slices from vehicle mice (up).

Encouraged by the ability of **HeckGal** to detect cellular senescence *in vitro*, we took a step forward and studied the potential of the **HeckGal** probe to detect cellular senescence *in vivo* in two different disease models of senescence: (i) BALB/cByJ female mice bearing 4 T1 breast cancer tumors treated with palbociclib and (ii) C57BL/6 J male mice with renal fibrosis induced by treatment with folic acid (FA). BALB/cByJ female mice were orthotopically injected in the mammary fat pad with 4 T1 cells (0.5 × 10^6^ cell/mouse) in order to generate breast tumors. Seven days later, palbociclib was administered daily by oral gavage to arrest tumor growth and induce cellular senescence. One week after, palbociclib treatment was started, 100 μL of **HeckGal** was injected intraperitoneally (i.p.) at a concentration of 13.3 mg/mL, and mice were sacrificed 3 h after treatment. Different organs (i.e., lungs, liver, kidney, and spleen) and tumors were harvested. Cellular senescence in palbociclib-treated tumors was assessed by SA-β-Gal staining (Figure 3A). The reduction of Ki67, a proliferative marker, observed by immunohistochemistry (IHC) was also indicative of cellular senescence in palbociclib-treated tumors (Figure 3B). Figure 3G shows the quantification of the Ki67 signal. *Ex vivo* IVIS images demonstrated that no fluorescent signal was observed in control animals, neither in tumors nor in lungs, liver, kidney, or spleen (Figure 3C–F), either in the presnce or absence of **HeckGal**. Tumors of mice treated with palbociclib in the absence of **HeckGal** were used to monitor tissue autofluorescence, and they displayed a weak emission. In contrast, tumors from mice previously treated with palbociclib and i.p. injected with **HeckGal** showed a strong emission signal in IVIS images (Figure 3F). Quantification of the average radiance intensity from organs and tumors was determined for each condition (Figure 3H). An emission enhancement of ca. 4.6-fold was observed in tumors treated with palbociclib when compared to control tumors. These results demonstrate that **HeckGal** is a potent tool to visualize senescence in a breast cancer tumor model treated with senescence-inducing therapy. Moreover, to evaluate **HeckGal** penetrability and their ability for two-photon imaging of senescent cells in the depth of tissues, fluorescence intensities of tumour slices from vehicle and palbociclib-treated mice at different depths were measured by a Z-scan model (Figure 3I,J). As could be seen in Figure 3I,J, a marked emission intensity was observed for the palbociclib-treated tumors administered with the **HeckGal** probe, and senescent cells could be visualized up to a depth of 150 μm. These results clearly indicated the ability of the **HeckGal** probe for tracking β-Gal activity at different depths using two-photon microscopy.

**Figure 4:**
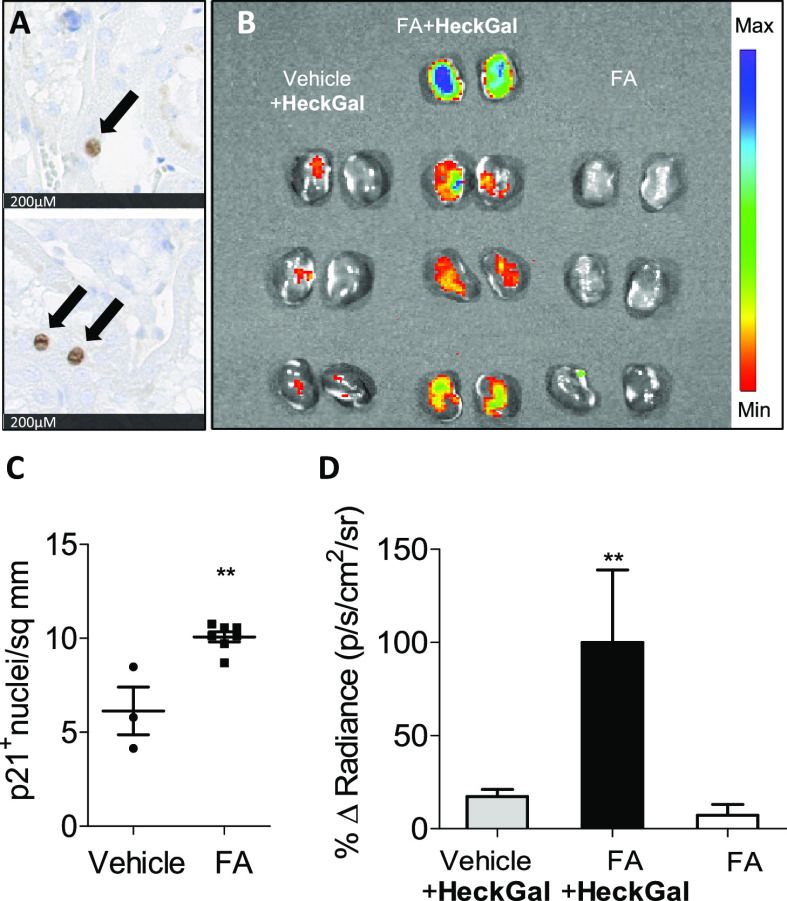
(A) Immunostaining for p21 in kidney slides. (B) IVIS images of kidneys from mice with renal fibrosis induced by FA treatment. From left to right: Vehicle mice + **HeckGa**l (6 mg/mL, 200 μL), FA-treated mice (with renal fibrosis) + **HeckGal** (6.6 mg/mL, 200 μL), and FA-treated mice (with renal fibrosis). Mice were sacrificed 5 h post-**HeckGal** injection. (C) Quantification of the p21 signal in paraffin sections of kidney from vehicle and FA-treated mice. Error bars represent SEM. (D) Quantification of average radiance intensity from kidneys showed in the 4B image. Error bars represent SEM (*n* = 3 for control mice treated with the probe and FA-treated mice, and *n* = 4 for FA-treated mice + **HeckGal**).

To assess the versatility of the probe, **HeckGal** was also tested to detect cellular senescence in a renal fibrosis model. For this purpose, C57BL/6 J male mice were i.p. injected with a single dose of 250 mg/kg of FA in order to generate renal fibrosis. Thirty-four days post-FA injection, the presence of cellular senescence in the kidneys was evaluated with p21 IHC immunostaining. An increase in the p21 signal was observed in the kidneys of FA-treated mice, confirming cellular senescence (Figure 4A). Figure 4C shows the quantification of positive p21 nuclei. Once cellular senescence was assessed in this model, 200 μL of **HeckGal** were i.p. injected at a concentration of 6.6 mg/mL (DMSO 1% corn oil).

Mice were euthanized 5 h post-**HeckGal** treatment, kidneys were excised and analyzed by IVIS imaging. Kidneys from control mice treated with **HeckGal** presented a very weak fluorescent signal (Figure 4B, left), whereas kidneys from FA-treated mice and injected with **HeckGal** (Figure 4B, middle) exhibited an intense emission (5.8-fold higher). FA-treated mice did not present any significant auto-fluorescence in the absence of the **HeckGal** probe (Figure 4B, right). Figure 4D showed the quantification of average radiance intensity from kidneys showed in 4B images.

## Conclusions

In summary, we report herein the synthesis of a new two-photon fluorescent probe for the detection of cellular senescence *in vivo*. **HeckGal** is based on a naphthalimide core linked to acetylated galactose that quenches the emission of **Heck** fluorophore. **HeckGal** is hydrolysed into the highly fluorescent **Heck** fluorophore in the presence of the β-Gal enzyme. *In vitro* detection of cellular senescence using **HeckGal** was assessed in senescent SK-Mel-103, A549, 4 T1, and BJ cell lines, in which senescence was induced by treatment with different therapies. The probe was validated to detect cellular senescence by one-photon and by two-photon confocal images and by FACS. The use of **HeckGal** to detect cellular senescence was also validated *in vivo* in BALB/cByJ mice bearing 4 T1 breast tumors, where senescence was induced with palbociclib. *Ex vivo* IVIS images showed that fluorescence ascribed to the hydrolyzed **HeckGal** probe (**Heck** fluorophore) was only observed in senescent tumors, whereas a negligible emission was found in other organs. Besides, **HeckGal** probe was also tested in a renal fibrosis model induced with FA. In this model, emission was only observed in fibrotic senescent kidneys from FA-treated mice. We hope that the studies presented here will help in the field of cellular senescence diagnosis in more translatable *in vivo* models. We also envisage that **HeckGal** or similar probes can be essential tools in the detection of senescent cells in aged or damaged tissues and to assess treatment response of senolytics in aging-related diseases.

## Supporting Information Available

Chemical characterization of the probe and reaction intermediates, experimental procedures, studies of the mechanism of hydrolysis, studies of fluorescence emission vs pH, and calculations of quantum yields; toxicity of **HeckGal** and **Heck** in SK-Mel-103 and 4 T1 cells as well as some confocal images of SK-Mel-103 with different confocal objectives; the immunohistochemical detection of Ki67 (PDF)
