# Supplementary material for: A Two-Photon Probe Based on Naphthalimide-Styrene Fluorophore for the In Vivo Tracking of Cellular Senescence
Source: Anal Chem. 2021 Jan 27;93(5):3052–60. doi: 10.1021/acs.analchem.0c05447 (PMC8719760; doi:10.1021/acs.analchem.0c05447)
Supplement: Supplementary file 1 — ac0c05447_si_001.pdf [file ac0c05447_si_001.pdf]

Supporting Information

A two-photon probe based on naphthalimide-styrene fluorophore for the *in vivo* tracking of cellular senescence

Beatriz Lozano-Torres,[1,2,3,4] Juan F Blandez,[1,2,4] Irene Galiana,[1,2,3,4] José A Lopez-Dominguez,[5] Miguel Rovira,[5] Marta Paez-Ribes,[6] Estela González-Gualda,[6] Daniel Muñoz-Espín,[6] Manuel Serrano,[5,7] Félix Sancenón[1,2,3,4]* and Ramón Martínez-Máñez[1,2,3,4]*

[1] Instituto Interuniversitario de Investigación de Reconocimiento Molecular y Desarrollo Tecnológico (IDM), Universitat Politècnica de València-Universitat de València, Camí de Vera S/N, Valencia, 46022 Spain.
[2] Unidad Mixta UPV-CIPF de Investigación en Mecanismos de Enfermedades y Nanomedicina, Universitat Politècnica de València, Centro de Investigación Príncipe Felipe, C/ Eduardo Primo Yúfera 3, Spain Valencia, 46012, Spain.
[3] CIBER de Bioingeniería, Biomateriales y Nanomedicina (CIBER-BBN), Av. Monforte de Lemos, 3-5. Pabellón 11. Planta 0, Madrid, 28029, Spain
[4] Unidad Mixta de Investigación en Nanomedicina y Sensores. Universitat Politècnica de València, IIS La Fe, Av. Fernando Abril Martorell, 10, Torre A 7ª planta, Valencia, 46026, Spain.
[5] Institute for Research in Biomedicine (IRB Barcelona). Barcelona Institute of Science and Technology (BIST), Carrer de Baldiri Reixac, 10, Barcelona, 08028, Spain.
[6] CRUK Cancer Centre Early Detection Programme, Department of Oncology, University of Cambridge, Hutchison/MRC Research Centre, Box 197, Cambridge, CB2 0XZ, UK
[7] Catalan Institution for Research and Advanced Studies (ICREA), Passeig Lluís Companys 23, 08010Barcelona, Spain
*Correspondence: rmaez@qim.upv.es

Supporting information

Table of Contents:

**Table 1**	**S2**
**Synthesis of Heck and Heck-gal**	**S3-S10**
**Heck fluorescence emission not depends on pH**	**S10**
**Hydrolysis reaction of Heck-Gal into Heck fluorophore**	**S11**
**Heck and HeckGal quantum yield measurements**	**S11**
***In vitro* viability assays**	**S12**
**Emission spectrum of Heck after two-photon excitation**	**S13**
**Co-staining does not affect to Heck signal**	**S14**

Table S1. Recently published fluorogenic molecular probes for cellular senescence detection.

Probe	λ_{exc}	λ_{em}	Cell line	Celullar senescence induction	In vivo model	Senescence induction in vivo	Multiphot on	IVIS	Reference
AHGa	750 nm	540 nm	SK-Mel-103	Palbociclib	SK-Mel-103 xenograft	Palbociclib	YES	NO	Lozano-Torres, B. et al. *J. Am. Chem. Soc.* 2017, **139**, 8808
NIR-BG	680 nm	708 nm	MCF7	Camptothecin or Radiation	CT26 xenograft	Camptothecin	NO	YES	Wang, Y. et al. *Sci. Rep.* 2019, **9**, 2102
			HeLa		HeLa xenograft				
NIR-BG2	650 nm	709 nm	HeLa	Camptothecin	HeLa xenograft	Camptothecin	NO	YES	Liu, J. et al. *bioRxiv*. 2020, https://doi.org/10.1101/2020.03.27.010827
SRP	495 nm	545 nm	HUVEC	H_2O_2	-	-	-	-	Kim, E. J. et al. *Sens Actuators B Chem*, 2018, **274**, 194
CBT-β-Gal	365 nm	510 nm	HeLa	H_2O_2	-	-	-	-	Makau J. N. et al. *ACS Omega*, 2020, **5**, 11299
βGal-1-4	361 - 370 nm	426 - 480 nm	A375	Hydroxyurea	-	-	-	-	Filho, M. S, et al. *Analyst*, 2018,**143**, 2680
			HT-29						
HeckGal	950 nm	550 nm	SK-Mel-103	Palbociclib	4T1 orthotopic breast tumour	Palbociclib	YES	YES	This work
			4T1						
			A549	Cisplatin	Renal fibrosis	Folic acid			
			SK-Mel-103	Doxorubicin					
			BJ fibroblast						

Synthesis of Heck and HeckGal.

6-Bromo-2-methoxy-1H-benzo[de]isoquinoléine-1,3(2H)-dione (**1**): A mixture of 4-bromo-1,8-naphthalic anhydride (2.7 g, 10 mmol) and methoxylamine hydrochloride (1.25 g, 15 mmol) were dissolved in anhydrous dioxane (150 mL). Then the system was purged with argon and trimethylamine was added (15 mL), maintaining the mixture overnight. The product was precipitated with cool water, filtered in vacuum and washed with cold water. The product was obtained as a yellow-brown solid 2.97 g. Yield 98%.^{1}H NMR (400 MHz, CDC_{13}) δ 8.71 (dd, J = 7.3, 1.1 Hz, 1H), 8.62 (dd, J = 8.5, 1.1 Hz, 1H), 8.46 (d, J = 7.9 Hz, 1H), 8.07 (d, J = 7.9 Hz, 1H), 7.88 (dd, J = 8.5, 7.3 Hz, 1H), 4.12 (d, J = 4.8 Hz, 3H). ^{13}C NMR (101 MHz, $CDCl_3$) δ 160.39 (2C), 134.18 (1C), 132.71 (1C), 131.80 (1C), 131.47 (1C), 131.31 (2C), 131.03 (1C), 128.41 (1C), 123.40 (1C), 122.51 (1C), 64.51 (1C).

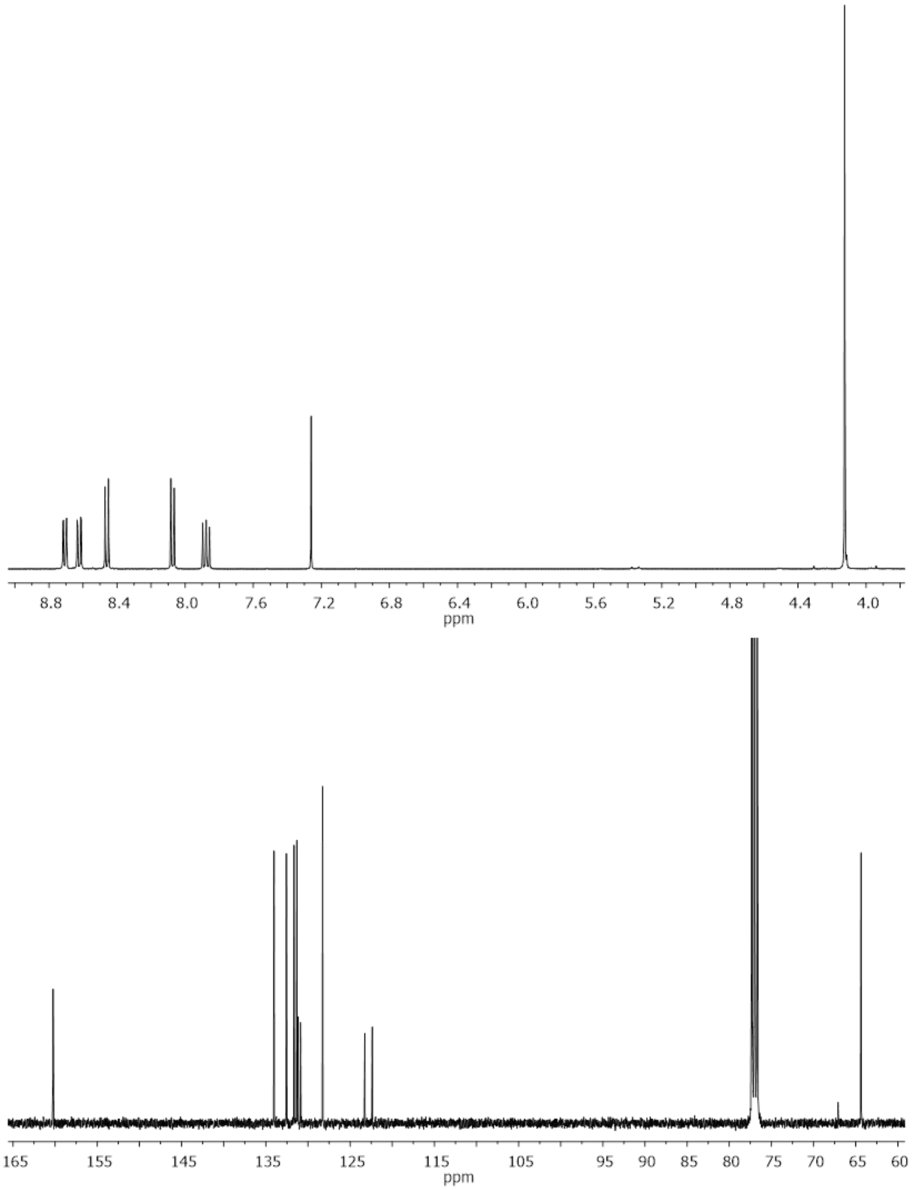

Figure S1. ^{1}H-NMR and ^{13}C-NMR of compound **1**.

4-{[(2-Methyl-2-propanyl)(diphenyl)silyl]oxy}benzaldehyde (**2**): A mixture of 4-hydroxybenzaldehyde (3 g, 24.6 mmol) and imidazole (1.68 g, 28.56 mmol) was added in a round bottom flask and purged with argon. The mixture was dissolved in anhydrous DMF (40 mL) and stirred at room temperature until the complete dissolution of the reagents. Then, tert-butylchlorodiphenylsilane was added dropwise (5.12 mL, 19.64 mmol) and the reaction was stirred for 4 h at room temperature. After complete reaction, the DMF was partially removed under vacuum. The reaction mixture was poured into water (50 mL) and the product was extracted with petroleum ether (2 x 50 mL). The organic layer was washed with brine (3 x 20 mL) and dried over $MgSO_4$. The petroleum ether was removed under vacuum and the product was obtained as a white solid, 6.75 g. Yield 76%. ^1H NMR (400 MHz, $CDCl_3$) δ 9.81 (s, 1H), 7.73 – 7.68 (m, 4H), 7.65 (d, J = 8.7 Hz, 2H), 7.48 – 7.43 (m, 2H), 7.42 – 7.35 (m, 4H), 6.86 (d, J = 8.6 Hz, 2H), 1.12 (s, 9H). ^{13}C NMR (101 MHz, $CDCl_3$) δ 191.00 (1C), 161.35 (1C), 135.53 (6C), 132.09 (1C), 131.84 (4C), 130.40 (2C), 128.12 (2C), 120.43 (2C), 26.52 (3C), 19.61 (1C).

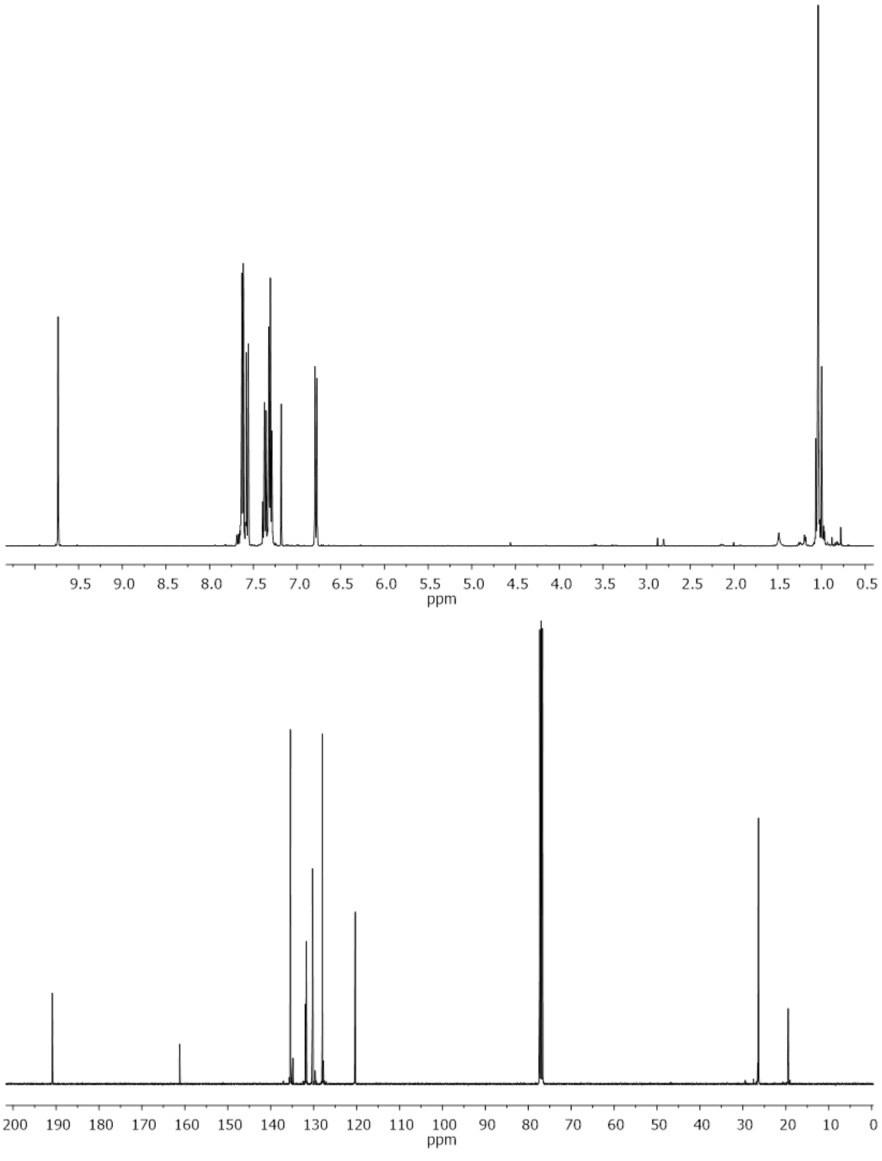

Figure S2. ^1H-NMR and ^{13}C-NMR of compound **2**.

4-{[(2-Methyl-2-propanyl)(diphenyl)silyl]oxy}styrene (**3**): Ph_3PCH_3I (6.4 g, 15.8 mmol) was added in a two-neck round bottom flash and then was purged with argon. Afterward, the solid was dissolved in anhydrous THF (40 mL) and a solution of n-BuLi was added dropwise (6.0 mL, 2.5 M in hexane, 15.8 mmol) at 0ºC. After stirring 15 min, a solution of compound **2** (3.67 g, 10.2 mmol) in anhydrous THF (20 mL) purged with argon was added dropwise. The mixture was stirred for 5 h and quenched with cold brine (50 mL). The product was extracted in diethyl ether (3 x 25 mL). The combined organic layers were dried over $MgSO_4$, filtered off and concentrated in vacuum. The crude product was purified in a silica flash column using diethyl ether as eluent. The product was obtained as a white solid, 3.06 g. Yield: 84 %. ^1H NMR (400 MHz, $CDCl_3$) δ 7.70 – 7.56 (m, 4H), 7.37 – 7.21 (m, 6H), 7.08 – 7.01 (m, 2H), 6.67 – 6.59 (m, 1H), 6.48 (dd, J = 17.6, 10.9 Hz, 1H), 5.43 (dd, J = 17.6, 1.0 Hz, 1H), 4.96 (dd, J = 10.9, 0.9 Hz, 1H), 1.01 (s, 3H). ^{13}C NMR (101 MHz, $CDCl_3$) δ 155.58 (1C), 136.44 (1C), 135.64 (2C), 133.01 (4C), 130.83 (1C), 130.06 (4C), 127.93 (2C), 127.30 (2C), 119.86 (2C), 111.71 (1C), 26.66 (3C), 19.61 (1C).

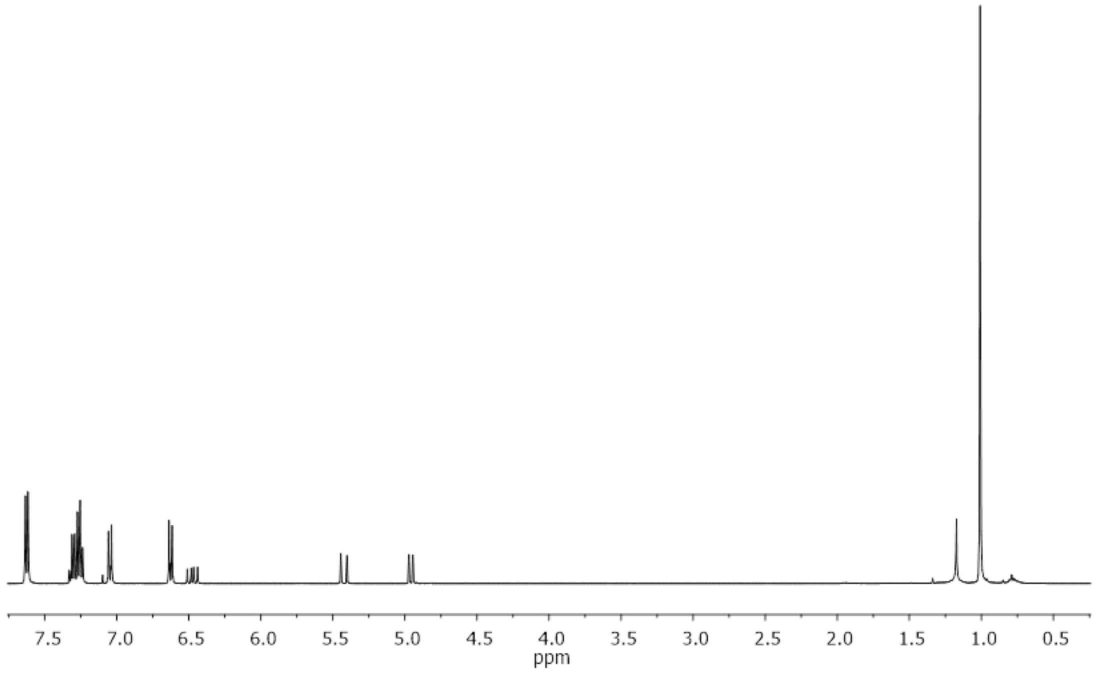

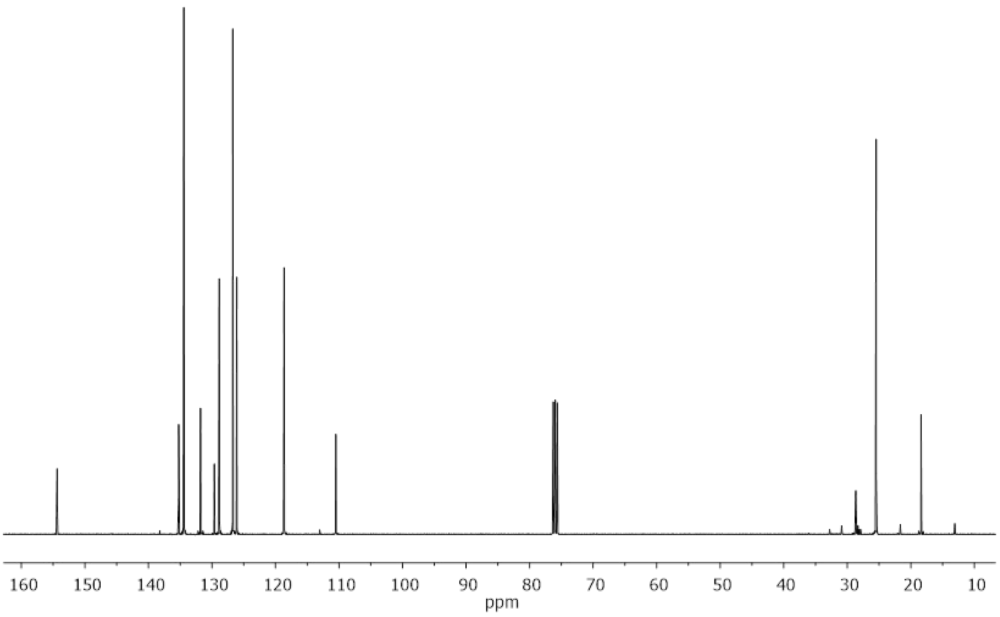

Figure S3. ^{1}H-NMR and ^{13}C-NMR of compound **3**.

E-2-(methoxyamine)-6-(4-hydroxystyryl)-1H-benzo[d]isoquinoleine-1,3(2H)-dione (**Heck**): A mixture of compound **2** (1.1 g, 3.1 mmol), **3** (1.1 g, 3.6 mmol), $Pd(AcO)_2$ (0.035 g, 0.15 mmol) and $P(o\text{-tolyl})_3$ (0.095 g, 0.312 mmol) were added into a two-neck round bottom flask. The system was purged with argon and the solids were dissolved in DMF (10 mL). After complete dissolution of the mixture, trimethylamine was added (2.17 g, 21.5 mmol). The mixture was heated at 90ºC for 24 h. After this time, the reaction was poured into cold water (20 mL) and the solid was washed with water and dried under vacuum. The solid was dissolved in THF and tetra-n-butylammonium fluoride was added (1.30 g, 5 mmol) to the solution. The mixture was stirred overnight at room temperature. The solvent was removed under vacuum and a brown solid was obtained. The crude product was purified by column chromatography using silica gel (eluent: dichloromethane/methanol 50:1) to obtain a red solid, 0.63 g. Yield 62 %. ^{1}H NMR (400 MHz, DMSO) δ 9.02 (dd, J = 8.6, 0,8 Hz, 1H), 8,56 (dd, J=7.3, 0.8 Hz, 1H), 8.49 (d, J = 7.9 Hz, 1H), 8.23 (d, J = 7.9 Hz, 1H), 8.01 (d, J = 16.1 Hz, 1H), 7.91 (dd, J = 8.6, 7.3 Hz, 1H), 7.71 (d, J = 8.6 Hz, 2H), 7.55 (d, J = 16.0 Hz, 1H), 6.85 (d, J = 8.6 Hz, 2H), 3.97 (s, 3H). ^{13}C NMR (101 MHz, DMSO) δ 160.23 (1C), 159.99 (1C), 158.49 (1C), 142.24 (1C), 135.69 (1C), 131.35 (1C), 131.01 (1C), 130.82 (1C), 129.29 (2C), 129.08 (1C), 127.73 (1C), 127.48 (1C), 126.88 (1C), 122.87 (1C), 122.82 (1C), 120.34(1C), 119.46 (1C), 115.67 (1C), 63.53 (1C). HRMS: Calculated for C21H15NO4 ($M^{+}H^{+}$) 346.1079 m/z; measured 346.1063 m/z ($M^{+}H^{+}$).

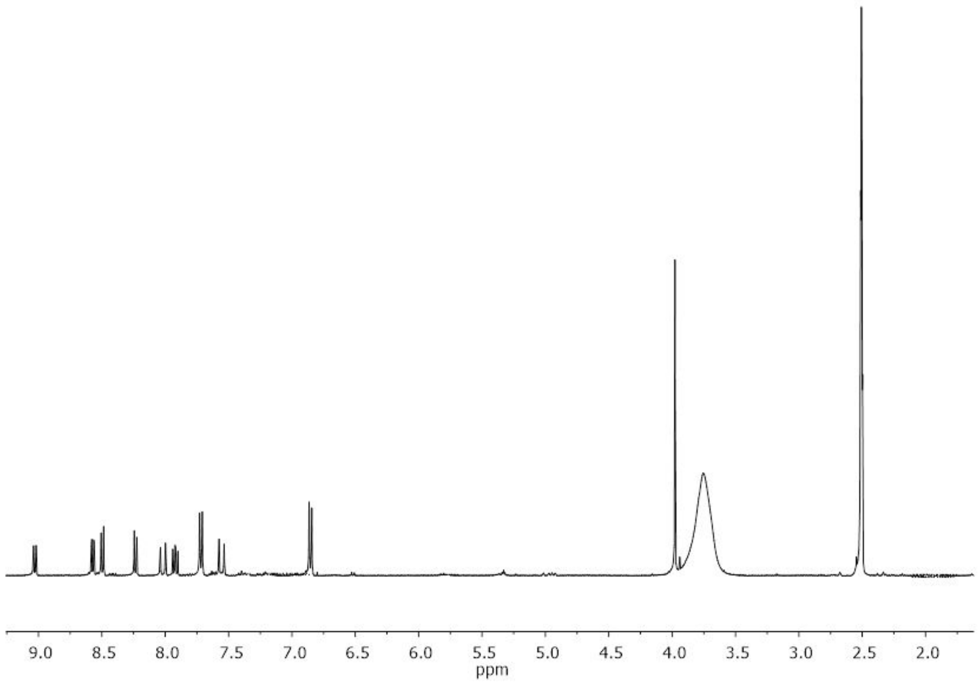
9.0
8.5
8.0
7.5
7.0
6.5
6.0
5.5
5.0
4.5
4.0
3.5
3.0
2.5
2.0
ppm

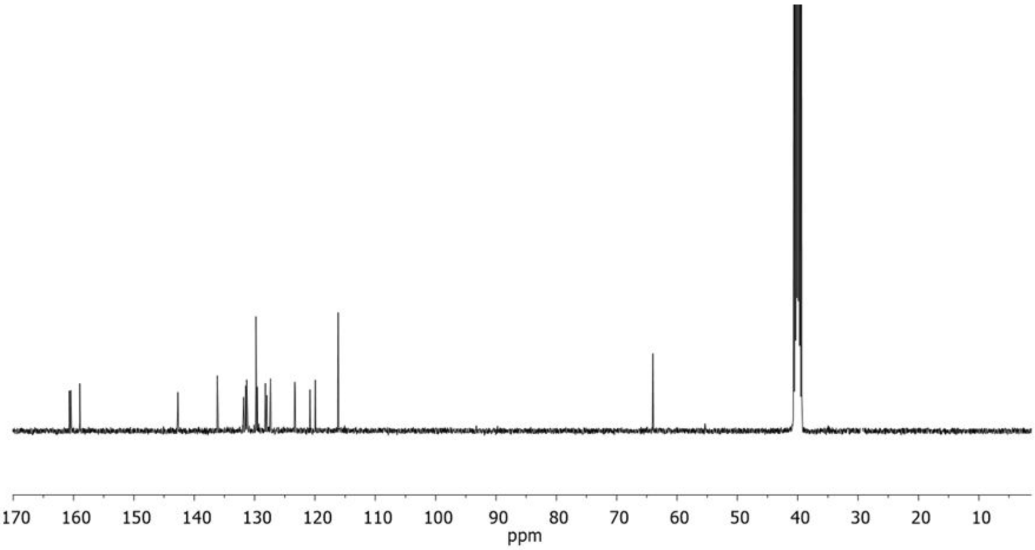
170
160
150
140
130
120
110
100
90
80
70
60
50
40
30
20
10
ppm

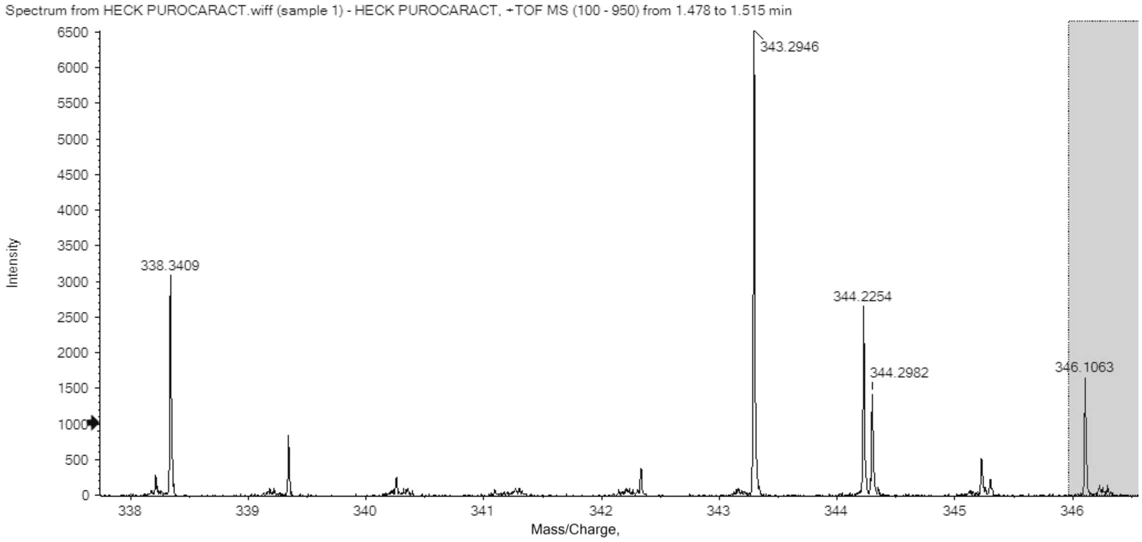

Figure S4. ^{1}H-NMR, ^{13}C-NMR and HRMS of **Heck** fluorophore.

E-2-(methoxyamine)-6-[4-(acetogalactoxy)styryl)]-1H-benzo[de]isoquinoleine-1,3(2H)-dione (**HeckGal**): A round bottom flask was charged with compound **Heck** (0.345 g, 1 mmol) and dissolved in methanol (10 mL). Then, NaOH (120 mg, 3 mmol) was added. The red color of the mixture changed to dark-red. The mixture was stirred for 1 h at room temperature. After complete reaction, the solvent was removed under vacuum pressure. Then, in a round bottom flask, acetobromo-α-D-galactose (822 mg, 2 mmol) and the solid obtained were mixed and the crude was purged with argon atmosphere. The mixture was dissolved in anhydrous acetonitrile. The reaction was stirred for 4 h at 70ºC and then the solvent was removed under vacuum pressure. The crude product was purified by column chromatography using silica gel (eluent: hexane/ethyl acetate 2:1) to obtain a red-orange solid 0.405 g. Yield 60 %. ^{1}H NMR (400 MHz, DMSO) δ 9.02 (dd, J = 8.6, 0.8 Hz, 1H), 8.57 (dd, J = 7.3, 0.8 Hz, 1H), 8.49 (d, J = 8.0 Hz, 1H), 8.23 (d, J = 8.0 Hz, 1H), 8.01 (d, J = 16.1 Hz, 1H), 7.91 (dd, J = 8.6, 7.3 Hz, 1H), 7.71 (d, J = 8.6 Hz, 2H), 7.55 (d, J = 16.1 Hz, 1H), 7.17 (d, J = 3.5 Hz, 1H), 6.85 (d, J = 8.6 Hz, 2H), 5.33 (dd, J = 1.1, 3.4 Hz, 1H), 5.28 – 5.19 (m, 2H), 4.90 (dd, J = 10.7, 3.5 Hz, 1H), 4.38 (t, J = 5.9 Hz, 2H), 3.97 (s, 3H), 2.14 – 1.90 (m, 12H). ^{13}C NMR (101 MHz, DMSO) δ 169.86 (1C), 169.80 (1C), 169.75 (1C), 169.09 (1C), 160.31 (1C), 160.09 (1C), 158.47 (1C), 142.55 (1C), 131.09 (1C), 129.20 (1C), 128.79 (1C), 128.62 (1C), 127.51 (1C), 126.54 (1C), 123.42 (1C), 122.95 (1C), 122.57 (1C), 121.76 (1C), 121.26 (1C), 120.64 (1C), 120.10 (1C), 119.33 (1C), 116.66 (1C), 115.73 (1C), 91.67 (1C), 69.79 (1C), 68.06(1C), 67.35 (1C), 64.32 (1C), 63.61 (1C), 61.08 (1C), 20.51 (1C), 20.42 (1C), 20.35 (1C), 20.29 (1C). HRMS: Calculated for $C_{35}H_{34}NO_{13}$ ($M^{+}H^{+}$) 676.2030 m/z; measured 676.2013 m/z ($M^{+}H^{+}$). Calculated for ($M^{+}K^{+}$) 714.1589; measured 714.1573 m/z ($M^{+}K^{+}$).

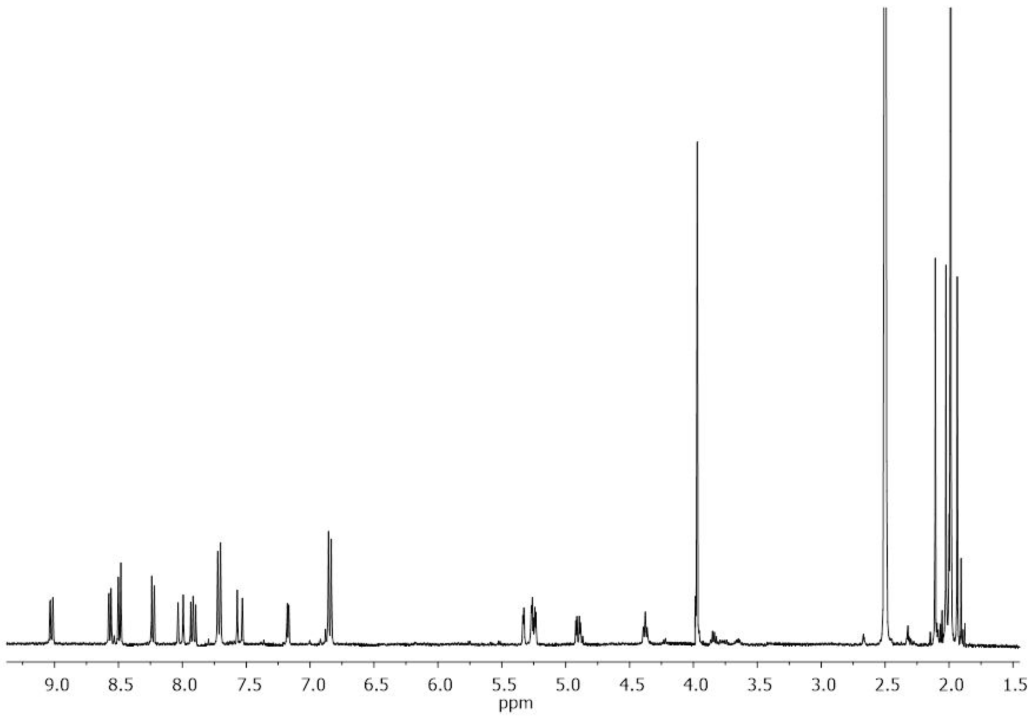
9.0 8.5 8.0 7.5 7.0 6.5 6.0 5.5 5.0 4.5 4.0 3.5 3.0 2.5 2.0 1.5
ppm

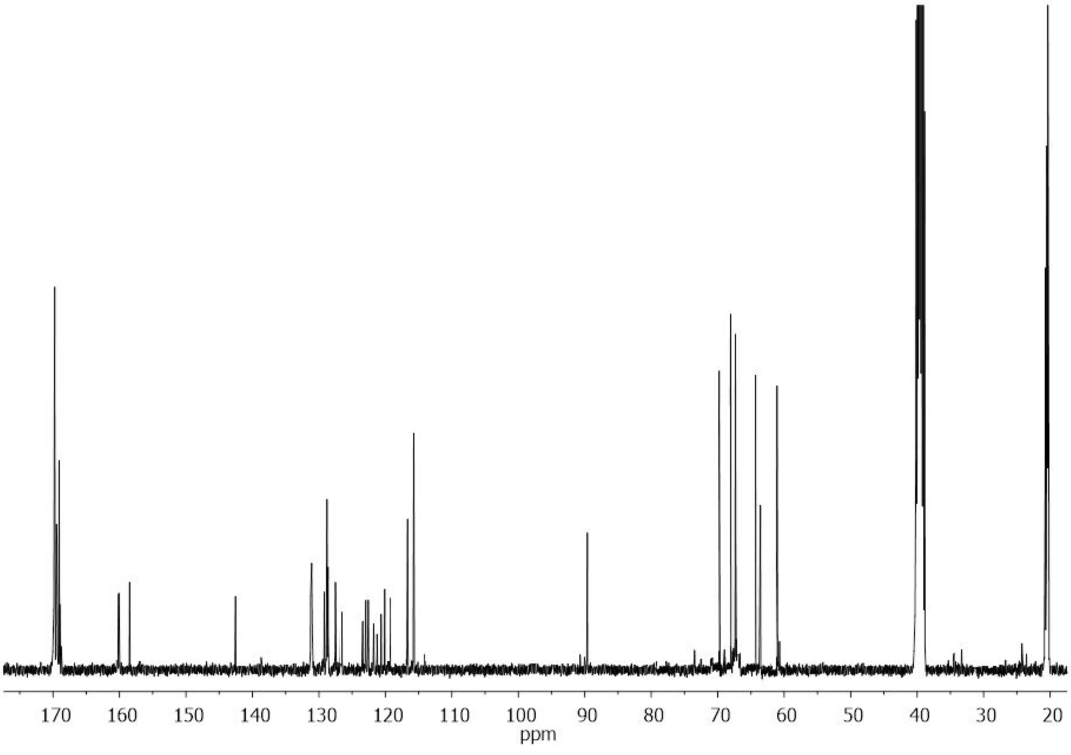
170 160 150 140 130 120 110 100 90 80 70 60 50 40 30 20
ppm

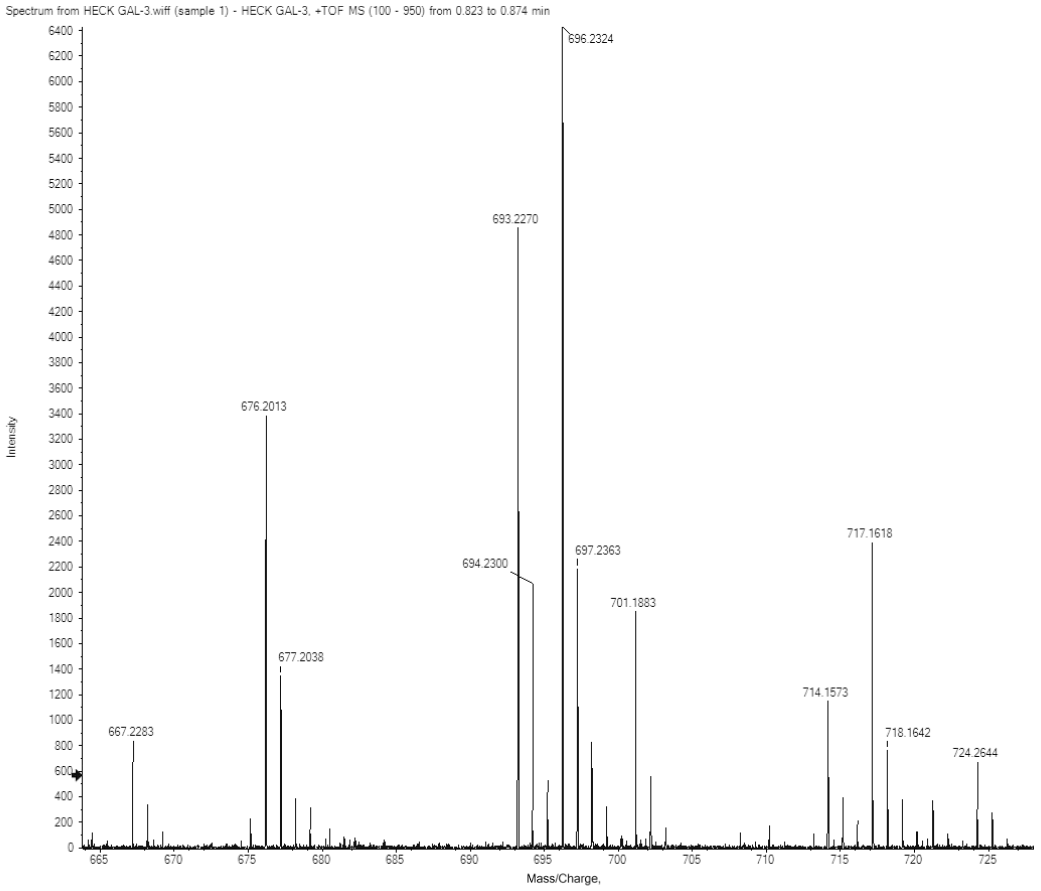

Figure S5. ^{1}H-NMR, ^{13}C-NMR and HRMS of **HeckGal** fluorophore.

Heck fluorescence emission not depends on pH

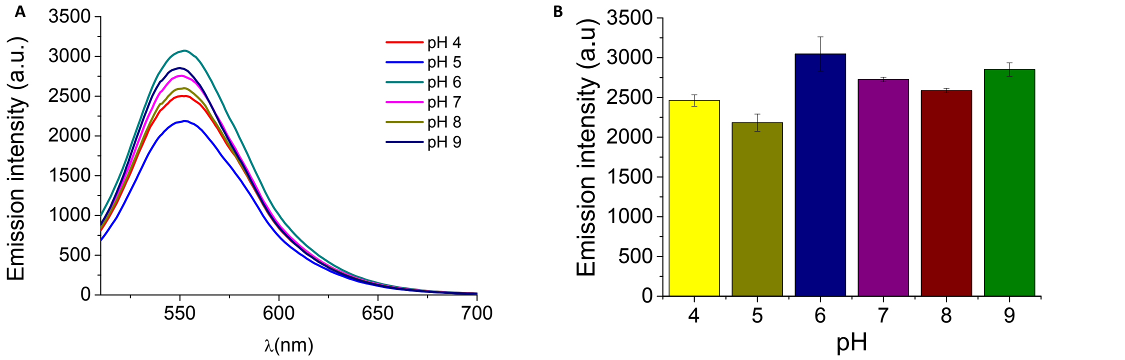

Figure S6. (A) Fluorescence spectra (λ_{ex} = 488 nm) of **Heck** (10^{-5} M) H_2O-DMSO (0.01%) at pH 4, 5, 6. 7, 8 and 9. (B) Emission intensity at 552 nm (λ_{ex} = 488 nm) of Heck (10^{-5} M) H_2O-DMSO (0.01%) solutions at pH 4, 5, 6, 7, 8 and 9. Error bars represent SD (n=3)

Hydrolysis reaction of Heck-Gal into Heck fluorophore

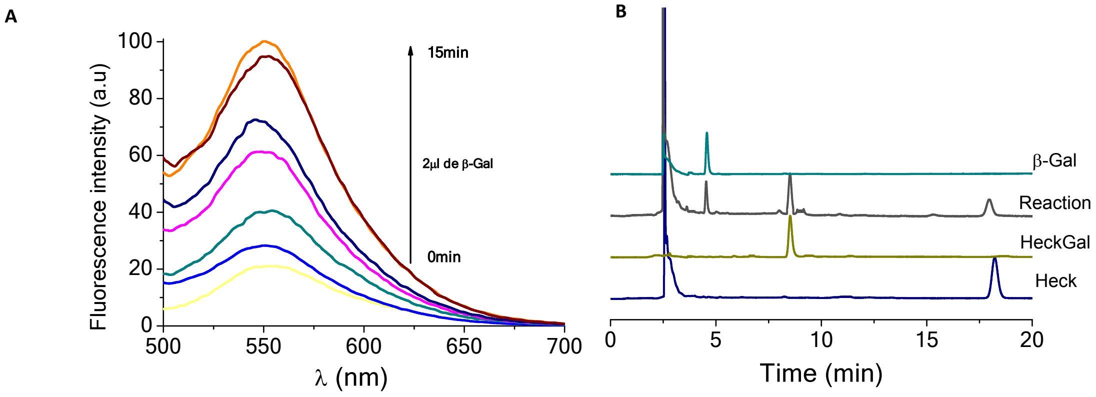

Figure S7. (A) Time dependence of fluorescence emission of **HeckGal** (yellow) aqueous solutions (pH 7)-DMSO (0.01%) in the presence of human β-Gal (λ_{ex} = 488 nm). The enhancement of emission band is ascribed to the conversion of **HeckGal** into **Heck** fluorophore (orange). (B) HPLC-UV studies of **HeckGal** aqueous solutions (pH 7)-DMSO (0.01%) hydrolysis in presence of human β-Gal.

Heck and HeckGal quantum yield measurements

Quantum yield values were measured with respect to rhodamine 6G as standard (Φ = 0.95) using the equation:

$$\frac{\Phi_x}{\Phi_s} = \frac{S_x}{S_s} \times \frac{1 - 10^{-A_s}}{1 - 10^{-A_x}} \times \frac{n_x^2}{n_s^2}$$

where x and s indicate the unknown and standard solution, respectively, Φ is the quantum yield, S is the area under the emission curve, A is the absorbance at the excitation wavelength and n is the index of refraction.

In vitro viability assays

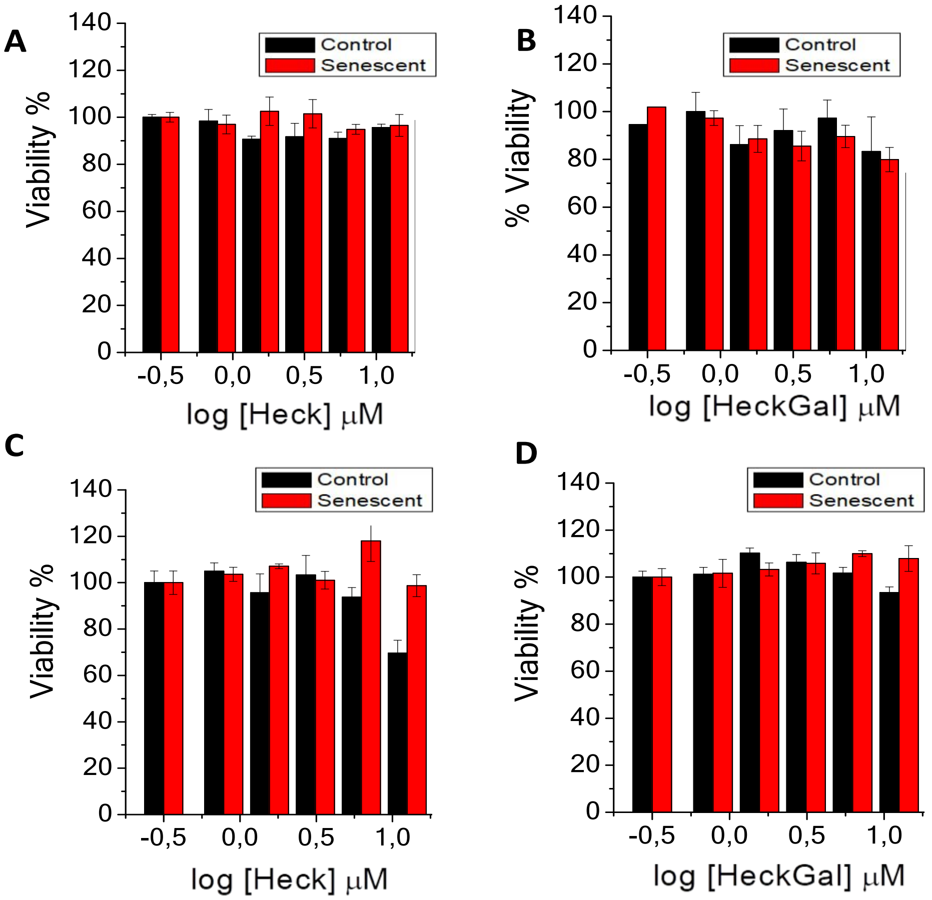

Figure S8. Relative *in vitro* viability in cultured cells. (A) Control (black) and senescent (red) SK-Mel-103 cells after incubation with **Heck** for 48 h at different concentrations. (B) Control (black) and senescent (red) SK-Mel-103 cells after incubation with **HeckGal** for 48 h at different concentrations. (C) Control (black) and senescent (red) 4T1 cells after incubation with **Heck** for 48 h at different concentrations. (D) Control (black) and senescent (red) 4T1 cells after incubation with **HeckGal** for 48 h at different concentrations. Note: both **HeckGal** and **Heck** have minimal toxicity and superior biocompatibility toward cultured cell line until 25 μM.

Emission spectrum of Heck after two-photon excitation

Fluorescence spectrum obtained from bright images of SK-Mel-103 cells treated with Palbociclib incubated with 10μM of **HeckGal** was measured with confocal microscope (OLYMPUS FV1000MPE) after two-photon confocal excitation. The obtained fluorescence spectrum corresponds to fluorescence spectrum of **Heck** fluorophore obtained in a two-photon fluorescence spectrophotometer.

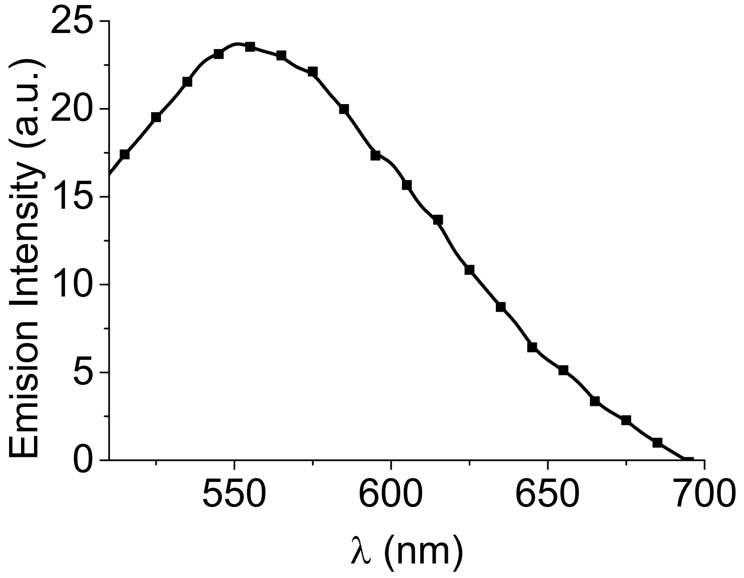

Figure S9. Fluorescence spectrum of **Heck** after confocal two-photon excitation.

Co-staining does not affect to Heck signal

In order to demonstrate that other typical staining kits do not affect to Heck fluorescence signal and the hydrolysis of **HeckGal**, confocal images from co-stained SK-Mel-103 with **HeckGal** (10µM) and Wheat Germ Agglutinin (WGA) stain (1 µg/ml).

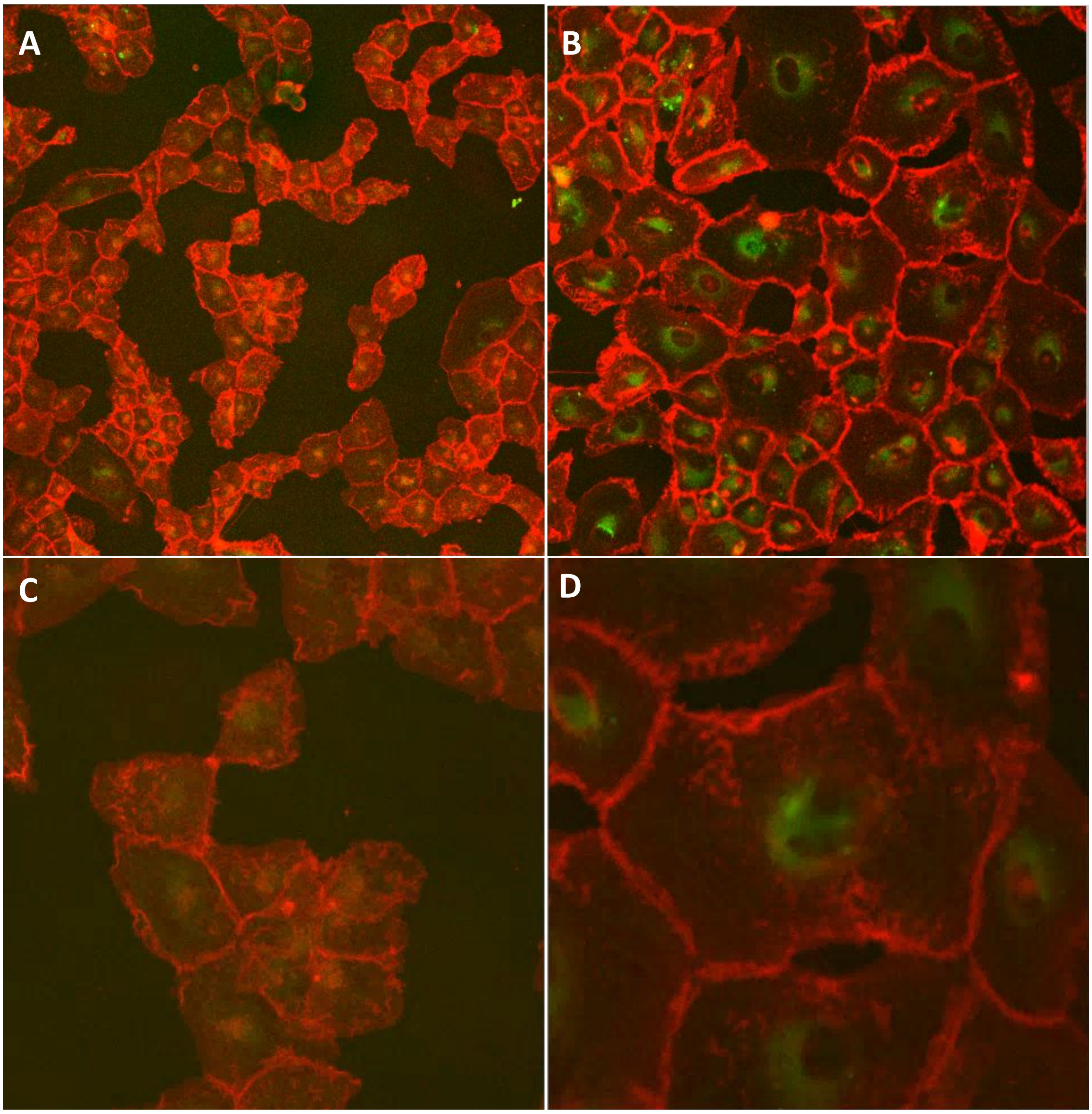

Figure S10. One photon confocal images of control SK-Mel-103 (A,C) in presence of 10 µM of **HeckGal** probe and Wheat Germ Agglutinin (WGA) stain (1 µg/ml) (Objectives: 20x a, 63x c) and SK-Mel-103 treated with palbociclib (B,D) in presence of 10 µM of **HeckGal** probe (Objectives: 20x b, 63x d).
